# Complications Following Local-Flap Lip Reconstruction After Tumor Removal: A Systematic Review

**DOI:** 10.7759/cureus.85155

**Published:** 2025-05-31

**Authors:** Sonal Kumar, Jamal Zahir, Mueen Megdadi, Lovingly M Ferrer Ocampo, Rachel MpanuMpanu, Zuivanna Rivas, Bhavana Thota, Laibah Ashfaq, Taylor E Collignon, Muhammad Q Zahir, George T Grace

**Affiliations:** 1 Plastic Surgery, Ross University School of Medicine, Miramar, USA; 2 General Surgery, Ascension Health, Baltimore, USA; 3 Plastic Surgery, Touro College of Osteopathic Medicine, Middletown, USA; 4 Plastic Surgery, Ross University School of Medicine, Miami, USA; 5 Plastic Surgery, University of California San Francisco, San Francisco, USA; 6 Plastic Surgery, UT Southwestern, Dallas, USA; 7 Plastic Surgery, University of Toronto, Toronto, CAN; 8 Internal Medicine, Lake Erie College of Osteopathic Medicine, Miami, USA; 9 Plastic Surgery, Research Division, Mile High MD, Denver, USA; 10 Plastic Surgery, Ascension Saint Agnes, Catonsville, USA

**Keywords:** aesthetic outcomes, flap complications, head and neck surgery, lip reconstruction, local flap reconstruction, oral cancer reconstruction, plastic surgery, reconstructive surgery, surgical outcomes, wound healing

## Abstract

Local flap reconstruction plays a critical role in managing defects following lip tumor removal. The aim of this review is to evaluate complications associated with local flap reconstruction for lip tumor removal. A systematic review was conducted utilizing the Preferred Reporting Items for Systematic Reviews and Meta-Analyses (PRISMA) guidelines. The PubMed (MEDLINE) database was queried from 2014 to 2024 with the following keywords: ("lip reconstruction" OR "cheiloplasty" OR "local flap") AND ("outcomes" OR "functional" OR "postoperative" OR "complications" OR "clinical"). Only retrospective and prospective studies, case series, randomized controlled trials, original research, and studies with patients requiring local flap reconstruction for tumor removal were included. We excluded studies that were related to cleft lip reconstruction, lip infection, burns, arteriovenous malformations (AVM), animal studies, technical reports, cadaveric studies, case reports, and review articles. After removal of duplicates and screening of titles, abstracts, and full-length text, 18 of the 749 initial articles were included in this review. The mean age across the articles was 66 ± 8 years (253 males; 124 females) with an average follow-up time of 23 ± 10 months. All of the studies included patients with squamous cell carcinoma (SCC), and only six of them analyzed patients with basal cell carcinoma (BCC). All studies included patients with bottom lip pathology, and only three with top lip pathology.

The mean complication rate was 8% across the studies. The most common reported complication was wound dehiscence (9/18), followed by paresthesias (7/18), microstomia (5/18), sialorrhea (4/18), and flap necrosis (2/18). The most common local flaps included: seven instances of Karapandzic, four instances of Abbe flaps, one case of Yu flap, two instances of step technique flaps, one case of Gillies flap, and two mentions of Webster flaps. Complications were reported in nine of the 19 articles. Local flap techniques provide favorable aesthetic and functional outcomes in lip reconstruction. However, current outcome measures vary widely, often including aesthetic evaluations, functional assessments, and complication rates, without standardized reporting. To enhance comparability and improve practice, surgeons should adopt standardized frameworks for reporting outcomes, such as validated tools for aesthetic and functional assessment (e.g., FACE-Q, PROMIS). Incorporating pre- and postoperative patient-reported outcomes, uniform complication classifications, and multi-center data collaboration will enable more consistent evidence-based decision-making and optimize patient care. Future studies should seek to utilize these practices for uniform reporting and improving patient outcomes.

## Introduction and background

Lip reconstruction plays a crucial role in restoring both function and aesthetics following trauma, congenital deformities such as cleft lip, and tumor excision. The lips are central not only to facial appearance but also to vital functions such as speech, eating, and facial expressions [1]. Over a 25-year study in a Northeast Brazilian population, 417 patients developed lip carcinoma, including 323 cases of squamous cell carcinoma and 94 cases of basal cell carcinoma [2]. Lip reconstruction, particularly when complex defects are involved, requires careful planning to balance these aesthetic and functional needs [3]. Over the years, various reconstructive techniques, including the Karapandzic, Abbe, and Estlander flaps, have been developed to achieve optimal outcomes, with each flap offering unique advantages based on the defect location, size, and patient characteristics [4]. Lip reconstruction is indicated for a variety of pathologies, including facial trauma, soft tissue defects after tumor excision, and cleft lip deformity [5]. The larger the defect, the more complex the reconstruction [6]. Likewise, large soft-tissue voids after tumor resection require complex reconstruction, with the objective of lip reconstruction being to ensure both aesthetic and functional improvements [7,8]. The goal of this narrative review was to summarize the growing literature on techniques, indications, and aesthetic and functional outcomes for lip reconstruction for trauma, tumor, and cleft lip. The recent advances in lip reconstruction techniques warrant an updated review. In conclusion, a thorough narrative review of reconstructive flap techniques for lip defects will provide surgeons with valuable insight into the most effective surgical approaches for different types of lip reconstruction, ultimately improving aesthetics and patient outcomes.

## Review

Methods

**Figure 1:**
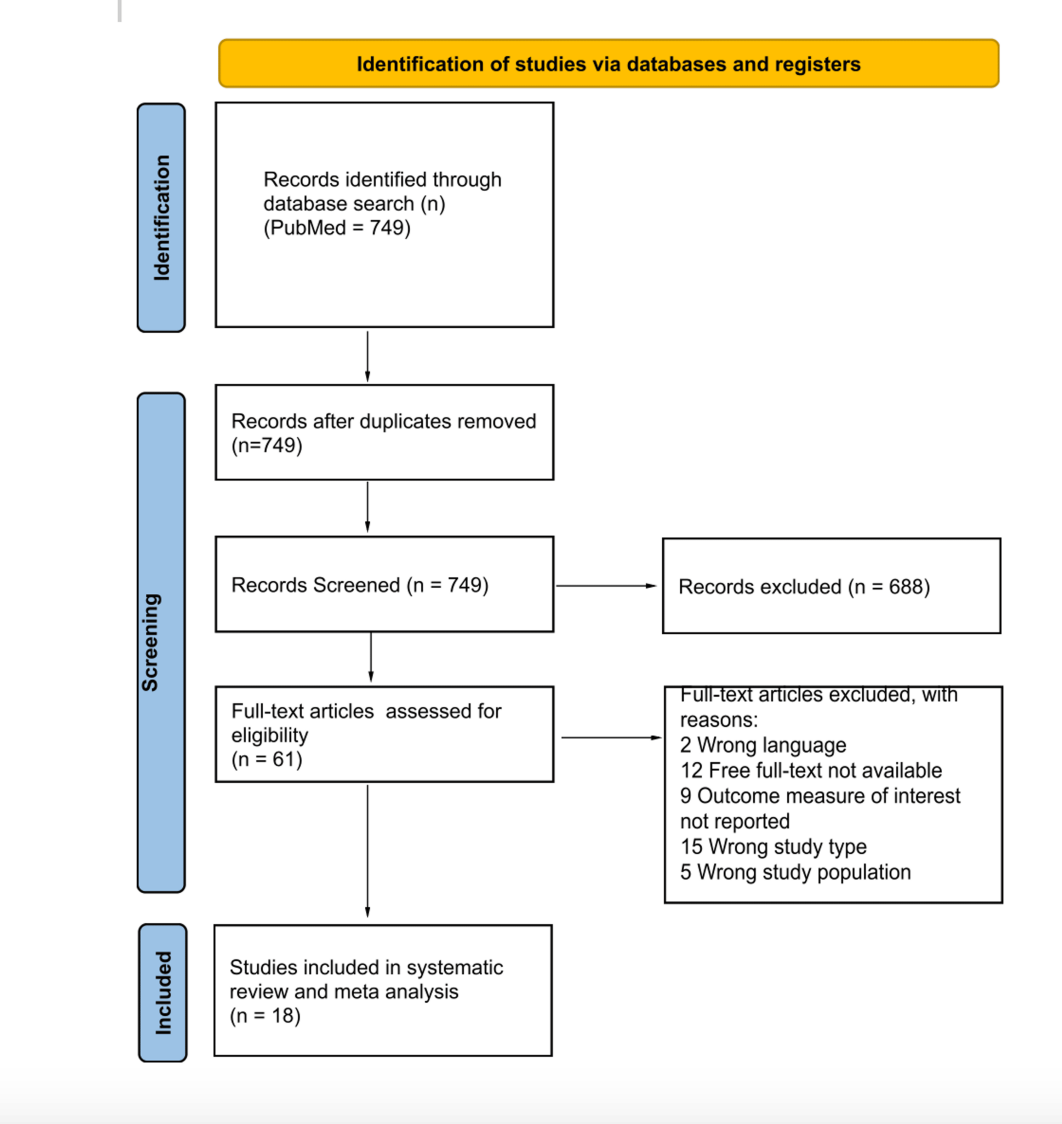
PRISMA Flow Diagram PRISMA: Preferred Reporting Items for Systematic Reviews and Meta-Analyses

We followed the Preferred Reporting Items for Systematic Reviews and Meta-Analyses (PRISMA) guidelines (Figure 1). We queried the PubMed (MEDLINE) database from 2014 to 2024 to develop this narrative review. Using the following keywords, we performed a Boolean search, which resulted in an initial list of 749 articles: ("lip reconstruction" OR "cheiloplasty" OR "local flap") AND ("outcomes" OR "functional" OR "postoperative" OR "complications" OR "clinical"). Zotero reference manager (Corporation for Digital Scholarship, Vienna, USA) was used to organize the retrieved literature and eliminate duplicates. Two independent reviewers screened the titles and abstracts for relevance. We also reviewed the citations of included studies for additional relevant articles.

The inclusion criteria were: only retrospective and prospective cohort studies, case series, randomized controlled trials, and original research. Only studies with individuals requiring lip reconstruction (e.g., after trauma and tumor excision) were included. All lip reconstruction techniques, including the Abbe flap for lip repair or the Estlander flap (modified Abbe flap), were studied. Only local flaps or flaps derived from the head and neck were considered for inclusion. Studies using either pediatric or adult populations were included. The studies must mention objective, measurable tools such as questionnaires and speech intelligibility tests to assess functional/clinical/aesthetic outcomes (e.g., speech, symmetry, scarring, visual analog scale (VAS) pain scores, etc.). Articles needed to report a follow-up period of at least 3 months to 1 year. We required the study to have a full free text available. We excluded studies that were related to cleft lip reconstruction, lip infection, burns, arteriovenous malformations (AVM), animal studies, technical reports, cadaveric studies, case reports, case series under five patients, conference abstracts, non-English language articles, and review articles. We recorded the author, year of publication, and title, among other data, from the included studies. Data regarding clinical, functional, and patient-reported outcomes were recorded and tabulated (Table 1). 

**Table 1:** Summary of data from 18 studies on lip carcinoma reconstruction BCC: basal cell carcinoma; SCC: squamous cell carcinoma; F: female; M: male

Total Studies	Value
Mean Age in Years (Mean ± SD)	66.18 ± 7.88
Follow-up in months (Mean ± SD)	23.1 ± 9.64
BCC (n (%))	6 (33.3%)
SCC (n (%))	18 (100%)
Upper lip (n (%))	3 (18.8%)
Lower lip (n (%))	16 (100%)
Sex (M/F) (n)	253/124
Complication Rate (%)	7.69%

Table 2 presents the common themes from the relevant articles [9-26].

**Table 2:** Summary of articles reviewed BCC: basal cell carcinoma; SCC: squamous cell carcinoma; F: female; M: male; NS: Not Specified

Author	Year	Study Design	Mean Age	Total (n)	Sex (n)	Type of Cancer	Upper or Lower Lip?	Type of Flap	Follow-up Time (months)	Complication Rate (%)	Complication Type
Fang et al [9]	2014	Prospective	70.5	12	8M/4F	BCC/SCC	Lower	V-Y island advancement	34.5	-	-
Kumar et al [10]	2014	Prospective	60	10	6M/4F	SCC	Lower	Abbe, Estlander	NS	20	One patient with blunting or fullness of oral commissure, one patient with orocutaneous fistula
Akdemir et al [11]	2014	Prospective	62	17	11M/6F	SCC	Lower	Platysma	16	-	-
Tetik et al. [12]	2014	Retrospective	60.6	10	6M/4F	SCC	Lower	Fujimori	16.5	-	-
Ye et al. [13]	2014	Retrospective	71	17	13M/4F	BCC/SCC	Lower	Karapandzic	21.5	-	-
Denadai et al [14]	2015	Prospective	64.3	12	11M/1F	SCC	Lower	Webster	26.3	16.7	Two patients with dehiscence
Casañas Villalba et al. [15]	2016	Retrospective	68	17	15M/2F	BCC/SCC	Lower	Yu	31.12	5.88	-
Teemul et al. [16]	2016	Retrospective	73.4	65	27M/38F	BCC/SCC	Upper and Lower	Karapandzic	NS	-	-
Uglesic, et al. [4]	2018	Case series	66.6	5	5M	SCC	Lower	Karapandzic, Abbe	33.6	-	-
Demirdover et al. [17]	2019	Prospective	66.8	21	17M/4F	SCC	Lower	Fan	12.1	14	Three patients with mild dehiscence all improved
Chen et al. [18]	2019	Case series	63	19	17M/2F	BCC/SCC	Upper and Lower	Abbe	13.37	10.5	Two patients with flap necrosis
Şirvan et al. [19]	2019	Prospective	58.9	16	14M/2F	SCC	Lower	Depressor anguli oris composite flap	NS	-	-
Costa-Gonzalez et al. [20]	2021	Retrospective	77	9	7M/2F	SCC	Lower	Colmenero	30	44.44	Two patients with dehiscence and two with drooling with resolution at final follow-up
Cortese et al. [21]	2022	Case series	64.5	6	5M/1F	SCC	Lower	Goldstein-Robotti and buccinator	24	-	-
Shaikh et al. [22]	2022	Prospective	45.4	21	17M/4F	SCC	Lower	Karapandzic, Estlander, Step	6	-	-
Park et al [23]	2024	Retrospective	70.2	34	17M/17F	SCC	Upper and Lower	Karapandzic, Abbe, Estlander	35.2	38.2	Dehiscence (2), microstomia (5), paresthesias (7), drooling (2)
Russo et al. [1]	2024	Retrospective	81.5	78	49M/29F	BCC/SCC	Lower	Karapandzic, Estlander, Step, Fan, Webster	NS	2.6	-
Tsai et al. [8]	2024	Retrospective	67.5	8	8M	SCC	Lower	Nasolabial flap	NS	-	-

Quality Assessment

The methodological quality of the included non-randomized studies was evaluated using the Methodological Index for Non-Randomized Studies (MINORS) criteria. This tool assesses 12 items related to study design and reporting, each scored from 0 (not reported) to 2 (adequately reported), with a maximum possible score of 24 for comparative studies. Two independent reviewers performed the assessment, and any disagreements were resolved through discussion and consensus. The mean MINORS score across all included studies was 12.88, indicating a moderate level of methodological quality.

Results

A total of 18 studies met the inclusion criteria out of an initial 749 records identified through the database search (Figure 1). After removal of duplicates and screening of titles, abstracts, and full texts, 61 articles were assessed for eligibility, with 43 excluded due to language restrictions, study design, lack of outcomes, or unavailable full text.

Across the 18 included studies, the mean patient age was 66.2 ± 7.9 years, with a total of 377 patients (253 males, 124 females). The average follow-up duration was 23.1 ± 9.6 months. All included patients underwent local flap reconstruction following excision of squamous cell carcinoma (SCC), while six studies (33.3%) also included patients with basal cell carcinoma (BCC). Lower lip defects were addressed in all 18 studies, whereas only three studies (16.7%) reported reconstruction of upper lip lesions.

The overall mean complication rate was 7.7%. The most frequently reported complication was wound dehiscence, documented in nine of the 18 studies. Other common complications included paresthesias (7/18 studies), microstomia (5/18), sialorrhea (4/18), and flap necrosis (2/18).

A variety of local flap techniques were employed. The Karapandzic flap was the most commonly utilized (seven studies), followed by the Abbe flap (four studies). Other techniques included the Estlander flap (three studies), step technique flaps (two studies), Webster flap (two studies), and single mentions of the Yu flap and Gillies flap.

Complications were reported in nine of the 18 studies, with variability in reporting methods and complication definitions. Several studies lacked standardized outcome measures, limiting direct comparisons across techniques.

Discussion

The findings of this study highlight important demographic and clinical characteristics of patients undergoing local flap reconstruction for lip tumor removal, as well as the associated complication rates. The predominance of older male participants with squamous cell carcinoma (SCC) aligns with the established epidemiology of lip malignancies, where SCC is the most common histologic subtype [1]. The higher prevalence of lower lip involvement compared to upper lip pathology is similarly consistent with prior literature, likely reflecting increased sun exposure and associated carcinogenic risk in the lower lip [2]. Despite the moderate overall complication rate of 7.7%, the variability in outcomes underscores the complexity of these reconstructions and the need for individualized surgical planning [3]. These results support the efficacy of local flap techniques while underscoring areas for improvement in postoperative outcomes and complication management [4].

The mean complication rate reported across studies was 7.7%, with wound dehiscence being the most frequently reported complication, followed by paresthesias, microstomia, and drooling [27]. Wound dehiscence, a common issue in surgical reconstructions, underscores the importance of careful flap design and meticulous postoperative care. Sialorrhea, or drooling, while less frequently reported, is also a significant concern, particularly in procedures involving the lower lip [20]. This complication can affect both the aesthetic and functional outcomes of lip reconstruction, influencing the patient's quality of life. The age and comorbidities of these patients should also be considered when evaluating the complication rates [21]. Older individuals may have decreased skin elasticity, impaired healing, or other underlying health issues that can increase the risk of surgical complications. This highlights the importance of tailored surgical planning and postoperative care in this population [22].

Among the various local flap techniques used for lip reconstruction, the most common were the Karapandzic and Abbe flaps, with seven and four instances, respectively [3]. These techniques are well-established for lower lip reconstruction due to their ability to provide well-vascularized tissue that can effectively close large defects while maintaining both function and appearance. The Karapandzic flap, which transposes the orbicularis oris muscle along with skin, is particularly useful for defects on the lower lip, allowing for functional restoration of lip closure [3]. The Abbe flap, often employed for defects in the central lower lip, provides an aesthetically pleasing result with minimal donor site morbidity [4]. Other flap techniques mentioned in the review, such as the Yu flap, step technique flaps, Gillies flap, and Webster flaps, were used less frequently [4,5]. While these techniques have been associated with positive outcomes in specific cases, their less frequent use suggests they may be reserved for more complex or less typical defects, or they may require additional expertise [5,6].

The Karapandzic and Abbe flaps, while commonly used for lip reconstruction, each have notable disadvantages. The Karapandzic flap carries a risk of microstomia, particularly for defects exceeding two-thirds of the lip, potentially limiting mouth opening and oral function [18,19]. It can also cause commissure blunting [9], affecting aesthetics, and may require significant cheek laxity for larger defects [20]. The Abbe flap necessitates a two-stage procedure, increasing patient inconvenience and the risk of infection or flap failure [21]. Additionally, it poses risks of donor site morbidity, such as scarring and functional impairment, and potential vascular compromise if the pedicle is damaged [22].

This study has several limitations that warrant consideration. First, the sample size is relatively small, which may limit the generalizability of the findings to broader populations. Additionally, the inclusion of various flap techniques, while reflective of clinical diversity, introduces heterogeneity that complicates direct comparison of outcomes. The lack of standardized reporting across studies further limits the ability to draw definitive conclusions about complication rates and reconstructive success. Furthermore, the underrepresentation of upper lip pathology highlights a potential selection bias and leaves a gap in understanding outcomes for this subset of patients. Future research should focus on larger, multicenter studies with standardized outcome measures to enable better comparisons across flap techniques. Moreover, prospective studies are needed to evaluate long-term functional and aesthetic outcomes, as well as to identify specific factors that may influence complication rates, such as tumor size, location, and patient comorbidities. Addressing these gaps will provide a more comprehensive understanding of local flap reconstruction in lip tumor management.

## Conclusions

Local flap reconstruction remains a cornerstone in the management of defects following lip tumor removal, offering favorable aesthetic and functional outcomes. While the mean complication rate of 7.7% highlights the challenges inherent to these procedures, wound dehiscence emerges as the most common complication. The diversity of flap techniques utilized underscores the adaptability of these methods to individual patient needs. However, the moderate methodological quality of the included studies, as assessed by the MINORS criteria (mean score 12.88), and variability in outcome reporting limit the strength of the conclusions and the ability to directly compare results across studies. Future research should prioritize standardized outcome measures, improved study design, and inclusion of a broader range of cases, particularly involving upper lip pathology, to further refine and optimize reconstructive strategies.
